# Structural characterizations and *α*-glucosidase inhibitory activities of four *Lepidium meyenii* polysaccharides with different molecular weights

**DOI:** 10.1007/s13659-023-00384-1

**Published:** 2023-06-06

**Authors:** Luan Wen, Zhou-Wei Wu, Li-Wu Lin, Abdulbaset Al-Romaima, Xing-Rong Peng, Ming-Hua Qiu

**Affiliations:** 1 State Key Laboratory of Phytochemistry and Plant Resources in West China, Yunnan Key Laboratory of Natural Medicinal Chemistry, Kunming Institute of Botany, Chinese Academy of Sciences, Kunming, 650201 Yunnan People’s Republic of China; 2 University of Chinese Academy of Sciences, Beijing, 100049 People’s Republic of China

**Keywords:** *Lepidium meyenii*, Polysaccharide, Molecular weight, Structural characterization, *α*-Glucosidase inhibition

## Abstract

Four polysaccharides (MCPa, MCPb, MCPc, MCPd) were obtained from *Lepidium meyenii* Walp. Their structures were characterized by chemical and instrumental methods including total sugar, uronic acid and protein content determination, UV, IR and NMR spectroscopy, as well as monosaccharide composition determination and methylation analyses. Four polysaccharides were a group of glucans with different molecular weights ranging from 3.12 to 14.4 kDa, and shared a similar backbone chain consisting of (1→4)-glucose linkages with branches attached to C-3 and C-6. Furthermore, bioactivity assay showed that MCPs had concentration-dependent inhibitory activity on *α*-glucosidase. MCPb (Mw = 10.1 kDa) and MCPc (Mw = 5.62 kDa) with moderate molecular weights exhibited higher inhibitory activity compared with MCPa and MCPd.

**Graphical Abstract:**

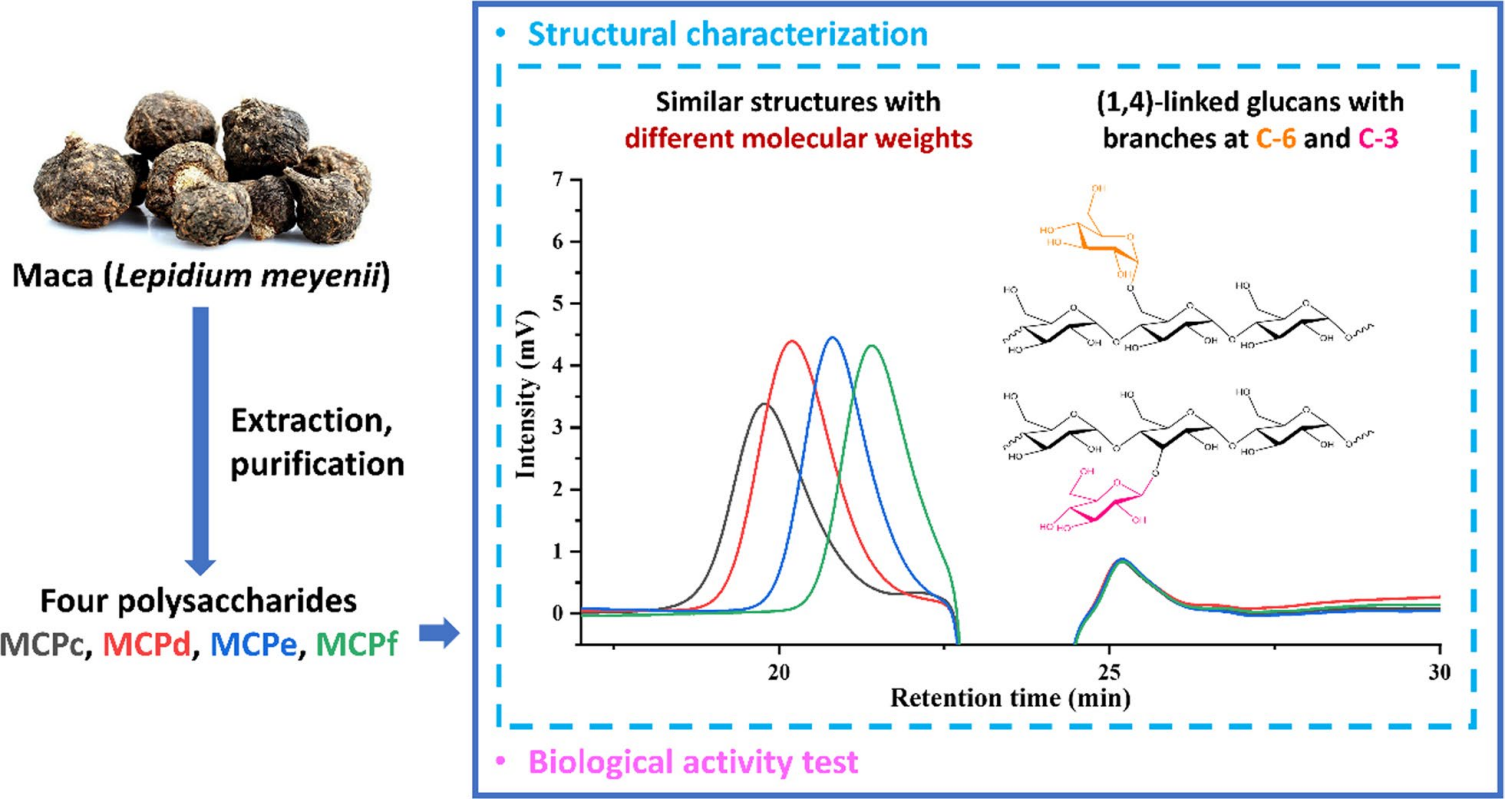

**Supplementary Information:**

The online version contains supplementary material available at 10.1007/s13659-023-00384-1.

## Introduction

Polysaccharides, which are one of the four major classes of biological macromolecules together with lipids, proteins and nucleic acids, are formed by more than 10 monosaccharide residues through glycosidic linkages [1]. In recent years, polysaccharides extracted from macrofungi, plants, animals and microorganisms have attracted increasing attention and are considered as ideal raw materials for functional food and drug because of their various activities, safety and fewer side effects [2]. Previous studies have reported that naturally obtained polysaccharides have a variety of biological activities, including anti-oxidation, immunomodulatory functions, anti-aging, anti-tumor, antiviral, anti-obesity, anti-bacterial [3, 4], and have therapeutic effects on human diseases such as Alzheimer’s disease, type 2 diabetes mellitus, and depression [3, 5]. These activities are reported to be strongly influenced by the structural properties of polysaccharides, including molecular weight distributions, monosaccharide residues, glycosidic linkages, and configurations [6].

*Lepidium meyenii* Walp., also known as Maca, is originally planted in the Andes region of Peru for more than 2000 years [7]. The tubers of *L. meyenii* have traditional applications in health promotion in South America, such as improvement of energy, sexual functions and reproductive functions, and therapeutic effects on osteoporosis, depression and anxiety [8]. Although the studies of *L. meyenii* mainly focused on small molecule compounds, saccharides are abundant in *L. meyenii*, accounting for more than half of the weight in dried *L. meyenii* products, including more than 30% polysaccharides, which might be significantly related to the edible properties of *L. meyenii* [9]. Previous studies reported the chemical composition of various *L. meyenii* polysaccharides, as well as biological activities such as immunomodulatory, anti-tumor, anti-oxidate, anti-bacteria and antiviral effects [10–20]. However, research on the structural characterization was still in its infancy, leading to the limitation of structure-function relationship studies.

In this study, four polysaccharides with different molecular weights have been isolated from *L. meyenii* roots. Furthermore, their structures have been characterized using chemical and instrumental methods, including total sugar, uronic acid and protein content determination, molecular weight determination, UV, IR and NMR spectroscopy, as well as monosaccharide composition determination and methylation analyses. Moreover, we investigated the *α-*glucosidase inhibition and immunomodulation of four polysaccharides.

## Results and discussion

### Extraction of MCPs

The crude polysaccharide was extracted by water extraction and ethanol precipitation from the roots of *L. meyenii.* After purification by gel column (Sephadex G-50 and Sephadex G-100) and ion exchange resin (DEAE-52), four homogeneous polysaccharides were obtained, and named as MCPa, MCPb, MCPc and MCPd, respectively.

### Total sugar, uronic acid and protein content determination of MCPs

The content of total sugar, uronic acid and protein of MCPs was determined by the phenol-sulfuric acid method [21], the carbazole-sulfuric method [22] and the Bradford method [23], respectively. The correlation coefficients (R^2^) of concentration-absorbance standard curves were higher than 0.99 (Additional file 1: Table S1). We substituted the absorbance of samples in the standard curves to calculate the content. Results (Table 1) showed that MCPd had the lowest content of total sugar (81.12%), and MCPc had the highest total sugar content (90.84%). Small amounts of uronic acid and protein have been identified in the four samples.

**Table 1:** Content of total sugar, uronic acid and protein of MCPs

Sample	Total sugar content (%)	Uronic acid content (%)	Protein content (%)
MCPa	87.03	0.79	1.23
MCPb	83.63	0.46	1.32
MCPc	90.84	ND	1.49
MCPd	81.12	ND	1.58

### Molecular weight determination of MCPs

The molecular weights of MCPs were determined using high-performance gel permeation chromatography (HPGPC). We drew a retention time-molecular weight standard curve with a series of dextran standards STD1-8 (Additional file 1: Figs. S1, S2) and then substituted the retention time of MCPs in HPGPC analyses (Additional file 1: Fig. S3) to calculate the molecular weights. Results (Table 2, Additional file 1: Fig. S4) demonstrated that MCPs were polysaccharides with different molecular weights, ranging from 3.12 kDa to 14.4 kDa. Homogeneity of MCPs was indicated by the polydispersity indexes (PDI) close to 1 and the molecular weight distributions (a normal distribution).

**Table 2:** Molecular weights and distributions of MCPs

Sample	Weight-average molecular weight (Mw, kDa)	Number-average molecular weight (Mn, kDa)	Polydispersity Index (PDI, Mw/Mn)
MCPa	14.4	11.4	1.26
MCPb	10.1	7.39	1.37
MCPc	5.62	4.31	1.30
MCPd	3.12	2.50	1.25

### UV spectroscopy of MCPs

Protein and nucleic acid have strong UV absorption at 280 nm and 260 nm, respectively [24]. The ultraviolet spectra of MCPs (Additional file 1: Fig. S5) showed no significant absorption signal peaks at above two wavelengths, indicating that the samples were free of protein or nucleic acid.

### Monosaccharide composition determination of MCPs

The MCPs were acid hydrolyzed and the obtained monosaccharides were derived by 1-phenyl-3-methyl-5-pyrazolone (PMP). Then the products were analyzed by HPLC. The monosaccharide compositions of MCPs were determined by comparing the HPLC spectrograms of mixed monosaccharide standards and the samples. Results (Additional file 1: Fig. S6) revealed that MCPa, MCPb, MCPc and MCPd mainly compose of glucose and they are a group of glucans with decreasing molecular weights.

### IR spectroscopy of MCPs

The IR spectra of MCPs had similar features (Additional file 1: Fig. S7), indicating that the samples contain similar functional groups with the only difference on their molecular weights. The absorbance bands at around 3412 cm^− 1^ and 2929 cm^− 1^ were the characteristic peaks of O–H stretching vibration and C–H stretching vibration respectively [25]. The peaks near 1635 cm^− 1^ were caused by the associated water [24]. The absorptions in the region of 1000 cm^− 1^ to 1200 cm^− 1^ were due to the C–O–C and C–O–H stretching vibration, indicating the existence of carbohydrates [26]. The peaks at 1154 cm^− 1^, 1081 cm^− 1^ and 1024 cm^− 1^ suggested the presence of a pyranose ring [24]. The absorbance bands at 930 cm^− 1^, 849 cm^− 1^ and 763 cm^− 1^ were the typical signals of an *α*-d-glucopyranose [27]. These signature signals indicated that MCPa, MCPb, MCPc and MCPd might have similar structures and are a group of *α*-d-glucans.

### Methylation analyses of MCPs

To determine the linkage types between the glycosidic residues, methylation–acetylation reactions were carried out and the products were analyzed by GC-MS. After the methylation of free hydroxyl groups, the polysaccharides were hydrolyzed into monosaccharides by acid. Then the exposed hydroxyl groups were acetylated to obtain partially methylated alditol acetates (PMAAs) [28, 29]. By identifying the structures of PMAAs in the reaction products based on GC-MS analyses, the linkage types of MCPs were determined. The GC chromatograms of methylation–acetylation products (Additional file 1: Fig. S8) showed one major peak at around 22.280 min, and three weak peaks at 20.146, 23.511 and 24.392 min. By comparing to the *m/z* signals of mass spectra in the Complex Carbohydrate Research Center (CCRC) spectral database for PMAAs, four peaks were respectively identified as 1,4,5-tri-*O*-acetyl-1-deuterio-2,3,6-tri-*O*-methyl-d-glucitol (Additional file 1: Fig. S9B), 1,5-di-*O*-acetyl-1-deuterio-2,3,4,6-tetra-*O*-methyl-d-glucitol (Additional file 1: Fig. S9A), 1,3,4,5-tetra-*O*-acetyl-1-deuterio-2,6-di-*O*-methyl-d-glucitol (Additional file 1: Fig. S9C) and 1,4,5,6-tetra-*O*-acetyl-1-deuterio-2,3-di-*O*-methyl-d-glucitol (Additional file 1: Fig. S9D), indicating the existence of 1,4-linked-d-glucopyranosyl residues, terminal-d-glucopyranosyl residues, 1,3,4-linked-d-glucopyranosyl residues and 1,4,6-linked-d-glucopyranosyl residues in MCPs. Furthermore, the molar ratio of the four residues in each sample was estimated from the peak areas (Table 3). These results indicated that four samples had a similar (1→4)-glucose backbone chain with some branches attaching to C-3 and C-6.

**Table 3:** Methylation analyses of MCPs

No	Retention time (min)	Glycosyl residue	Mass fragments (*m/z*)	Molar ratio			
				MCPa	MCPb	MCPc	MCPd
1	20.146	T-Glc*p*	59, 71, 87, 102, 118, 129, 145, 162, 175, 205	2.232	2.131	2.415	2.581
2	22.280	→4)-Glc*p*-(1→	59, 71, 87, 99, 118, 129, 142, 159, 173, 203, 233	9.153	14.469	8.943	13.231
3	23.511	→3,4)-Glc*p*-(1→	59, 71, 87, 98, 118, 129, 143, 160, 171, 185, 203, 231, 305	1.000	1.000	1.438	1.540
4	24.392	→4,6)-Glc*p*-(1→	59, 71, 85, 102, 118, 127, 142, 159, 187, 201, 261	1.213	1.069	1.000	1.000

### NMR analyses of MCPs

MCPs had similar ^1^H (Additional file 1: Fig. S10) and ^13^C NMR spectra (Additional file 1: Fig. S11), suggesting the similarity of their structures. To further elucidate the structural features, we took MCPc as an example. Chemical shifts were assigned (Table 4) according to 1D NMR (^1^H NMR and ^13^C NMR) and 2D NMR (COSY, TOCSY, ROESY, HSQC, HMBC) spectra (Additional file 1: Figs. S12–S18). ^1^H NMR spectrum (Additional file 1: Fig. S12) showed that the anomeric region contained four signals at *δ*_H_ 5.33, 5.28, 5.26 and 4.89, which were respectively assigned to →4)-Glc*p*(1→ (residue A), →3, 4)-Glc*p*(1→ (residue B), →4, 6)-Glc*p*(1→ (residue C) and T-Glc*p* (residue D). As *δ*_H_ 4.89–5.53 ppm was the region of *α*-anomeric proton [30], combined with the characteristic signals of *α*-d-glucopyranose in the IR spectra, the residues were considered to have *α*-configurations. The overlapping signals at the region of H-2 to H-6 (*δ*_H_ 3.2–4.1 ppm) were then assigned according to the ^1^ H-^1^ H COSY (Additional file 1: Fig. S14) and TOCSY (Additional file 1: Fig. S15) spectra, combing with ROESY (Additional file 1: Fig. S16), HSQC (Additional file 1: Fig. S17) and HMBC (Additional file 1: Fig. S18) spectra, as well as previous reports [24, 30, 31]. According to the assignment of anomeric protons and HSQC (Additional file 1: Fig. S17) spectrum, the anomeric carbon resonance at 99.8, 99.6 and 98.6 ppm in ^13^C NMR (Additional file 1: Fig. S13) spectrum was respectively assigned to residue B/C, residue A and residue D. The signals of C-2 to C-6 were then determined mainly based on ROESY (Additional file 1: Fig. S16), HSQC (Additional file 1: Fig. S17) and HMBC (Additional file 1: Fig. S18) spectra. The C-4 signals at around 76.7 ppm of residues A/B/C shifted to a lower field compared with residue D, indicating the existence of *O*-substituted C-4 of residues A/B/C. Similarly, the lower C-3 signal of residue B and the lower C-6 signal of residue C respectively suggested the presence of substituted groups on C-3 position of residue B and C-6 position of residue C.

**Table 4:** ^1^H and ^13^C NMR chemical shifts of MCPc in D_2_O

Glycosyl residue	Chemical shift (*δ*, ppm)						
		1	2	3	4	5	6
Residue A, →4)-Glc*p*(1→	H C	5.33 99.6	3.55 71.5	3.88 73.3	3.58 76.7	3.76 71.1	3.78, 3.69 60.5
Residue B, →3, 4)-Glc*p*(1→	H C	5.28 99.8	3.55 71.5	3.81 77.7	3.58 76.7	3.76 71.1	3.78, 3.69 60.5
Residue C, →4, 6)-Glc*p*(1→	H C	5.26 99.8	3.55 71.5	3.88 73.3	3.58 76.7	3.76 71.1	3.81, 3.69 67.6
Residue D, T-Glc*p*	H C	4.89 98.6	3.51 71.6	3.63 72.8	3.34 69.3	3.96 70.3	3.78, 3.69 60.5

**Fig. 1:**
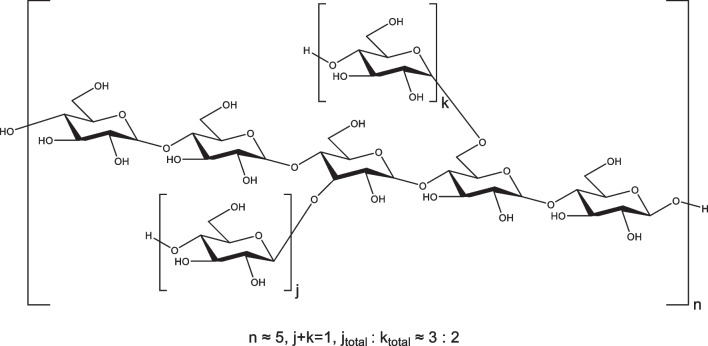
The predicted chemical structure of MCPc

The connection of residues was further determined according to ROESY (Additional file 1: Fig. S16) and HMBC (Additional file 1: Fig. S18) spectra. The prominent cross-peaks of *δ*_H_ 5.33 (H-1 of residue A)/*δ*_H_ 3.55 (H-4 of residues A/B/C) and *δ*_H_ 5.24–5.30 (H-1 of residues B/C)/*δ*_H_ 3.55 (H-4 of residues A/B/C) in ROESY spectrum (Additional file 1: Fig. S16A) and *δ*_H_ 3.58 (H-4 of residues A/B/C)/*δ*_C_ 99.2-100.2 (C-1 of residues A/B/C) in HMBC spectrum (Additional file 1: Fig. S18) indicated that residues A was linked at the C-4 position of residues B/C and residues B/C was also linked at the C-4 position of residue A. The weak cross-peaks of *δ*_H_ 4.89 (H-1 of residue D)/*δ*_H_ 3.81 (H-3 of residue B or H-6 of residue C) in ROESY spectrum (Additional file 1: Fig. S16B) and *δ*_H_ 3.81 (H-3 of residue B or H-6 of residue C)/*δ*_C_ 98.6 (C-1 of residue D) in HMBC spectrum (Additional file 1: Fig. S18) suggested that residue D was linked at the C-3 position of residue B and the C-6 position of residue C. These findings supported that the sequence of MCPc contained a (1,4)-linked glucan backbone chain and was partially substituted on C-3 and C-6 positions, consistent with the results of methylation analysis. The molar proportion of glycosyl residues of the backbone chain (residues A/B/C) and branched chains (residue D) was estimated by the ratio of peak area and was determined as around 5:1, which was also consistent with the results of methylation analysis. Furthermore, the molar ratio of O-substituted branches on C-3 and C-6 was around 3:2 according to the methylation analysis. Therefore, the predicted structure of MCPc was proposed, as shown in Fig. 1.

### Biological activities of MCPs

**Fig. 2:**
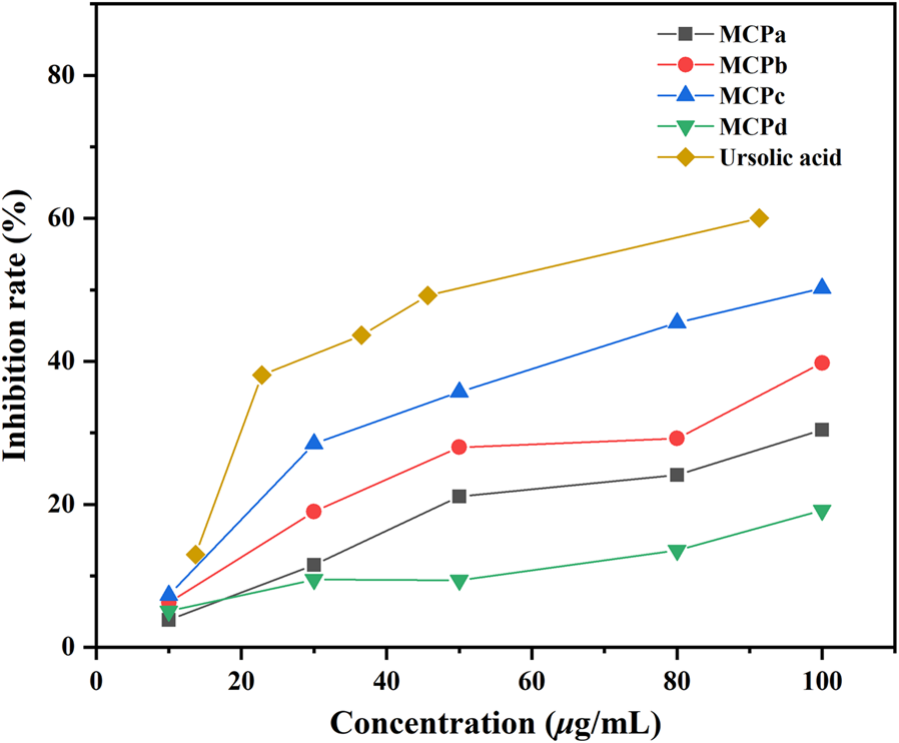
*α-*Glucosidase inhibitory activity of MCPs

The *α-*glucosidase inhibitory activity of MCPs was determined, and the results (Fig. 2) showed that the four samples exhibited concentration-dependent inhibitory activity on *α*-glucosidase. Furthermore, comparing with MCPa and MCPd, MCPb (Mw = 10.1 kDa) and MCPc (Mw = 5.62 kDa) with moderate molecular weights exhibited higher inhibitory activity, with the inhibition rates lower than that of the positive control (ursolic acid).

Our findings indicated that the *α*-glucosase inhibition of *L. meyenii* polysaccharides is closely related to their molecular weights, which is corresponding to the previous results [32–34]. It might be caused by the influence of molecular weight on the solubility of polysaccharides and the contact with receptor proteins.

## Experimental section

### Plant material

Rhizomes of *Lepidium meyenii* Walp. cultivated in Peru were obtained in July 2021 from LuoShiWan Traditional Chinese Medicine Market in Kunming, China. Samples were identified by the corresponding author of this paper (Professor Qiu). The specimen was kept in the State Key Laboratory of Phytochemistry and Plant Resources in West China, Kunming Institute of Botany, Chinese Academy of Sciences.

### Chemicals

Dextran standards STD1-8 (Mw: 2700, 5250, 9750, 13,050, 36800, 64,650, 135,350, 300,600 Da) were purchased from National Institute for Food and Drug Control (Beijing, China). Monosaccharide standards mannose (Man), rhamnose (Rha), arabinose (Ara), glucose (Glc), galactose (Gal), glucuronic acid (GlcA), galacturonic acid (GalA) and *N*-acetyl-glucosamine (GlcNAc) were provided by Yuanye Bio-Technology Co., Ltd. (Shanghai, China), fucose (Fuc) was purchased from Lemeitian Pharmaceutical Technology Co., Ltd. (Chengdu, China). PMP and *α*-glucosidase were obtained from Yuanye Bio-Technology Co., Ltd. (Shanghai, China). Sodium chloride, sodium hydroxide and glacial acetic acid were purchased from Kelong Chemical Co., Ltd. (Chengdu, China). Ammonium acetate, absolute ethyl alcohol, hydrochloric acid and phenol were obtained from Xilong Scientific Co., Ltd. (Guangzhou, China). Sulfuric acid was purchased from Sinopharm Chemical Reagent Co., Ltd. (Shanghai, China). Carbazole was purchased from Macklin Biochemical Co., Ltd. (Shanghai, China). Coomassie brilliant blue G-250 was obtained from Haohong Scientific Co., Ltd. (Shanghai, China). Iodomethane and deuterated sodium borohydride (NaBD_4_) were purchased from Anhui Zesheng Technology Co., Ltd. (Anqing, China). Bovine serum albumin (BSA), anhydrous dimethyl sulfoxide (DMSO), pyridine, *p*-nitrophenyl-*α*-d-glucopyranoside (PNPG) and ursolic acid were purchased from Aladdin Biochemical Technology Co., Ltd. (Shanghai, China). Phosphoric acid, acetic anhydride and trichloromethane (CHCl_3_) were obtained from Chuandong Chemical Co., Ltd. (Chongqing, China). Trifluoroacetic acid (TFA) was obtained from J&K Scientific Co., Ltd. (Beijing, China). Acetonitrile (mass spectrometry grade) was purchased from Oceanpak Alexative Chemicals Co., Ltd. (Gothenburg, Sweden). 95% ethanol, methanol and dichloromethane (CH_2_Cl_2_) were purchased from Yunnan Chemical Reagent Co., Ltd. (Kunming, China). The RAW264.7 murine macrophage cell line was purchased from National Collection of Authenticated Cell Cultures, Chinese Academy of Sciences (Shanghai, China). Dulbecco’s Modified Eagle Medium (DMEM) and new bovine calf serum were obtained from Biological Industries, Ltd. (Beit Haemek, Israel). Griess reagent and lipopolysaccharide (LPS) were obtained from Sigma Co., Ltd. (New York, USA).

### Extraction of MCPs

The roots of *L. meyenii* were cut into fine powder by a pulverizer. The obtained powder (1 kg) was extracted with 95% ethanol (v/v) to get defatted. The dried fat-free product was then mixed with pure water (1:8, w/v) at 80 ℃ for 3 h three times. The extracts were centrifuged (4000 rpm, 10 min), and the supernatant was collected and concentrated.

The obtained supernatant was then precipitated with 95% ethanol (v/v) to a final concentration of 40% and the mixture was kept overnight. After the centrifugation (4000 rpm, 10 min) of the mixture, precipitation (Fr. 1) and supernatant were obtained respectively. The separated supernatant was concentrated and precipitated by adding 95% ethanol (v/v) to a final concentration of 60% to obtain another precipitation (Fr. 2) and supernatant. Similarly, the separated supernatant was concentrated, then absolute ethanol was added to a final concentration of 80% to obtain precipitation (Fr. 3) and supernatant. The supernatant was dried by a rotary evaporator and named Fr. 4. Four obtained fractions were analyzed by HPGPC. We used an HPGPC apparatus (LC 20-AT, Shimadzu, Japan) equipped with a refractive detector (RID-20 A, Shimadzu, Japan) and a tandem gel column (OHpak SB-804 HQ, Shodex, Japan). The mobile phase was 0.1 M NaCl, the flow rate was 0.5 mL/min and the analysis time was 35 min. According to the analysis results, Fr. 2 was chosen to further separation.

We dissolved Fr. 2 (7.2 g) in pure water and then separated it by fractional ethanol precipitation as above. Fr. 2 was precipitated by 95% ethanol (v/v) to a final concentration of 40% to obtain the precipitation (Fr. 2 − 1), then the supernatant was concentrated and precipitated with 60% ethanol to obtain the precipitation (Fr. 2–2). Afterward, the obtained supernatant was similarly concentrated and precipitated with 80% ethanol, and the precipitation (Fr. 2–3) and the supernatant (Fr. 2–4) were collected respectively.

Fr. 2–2 (337 mg) was dissolved in pure water and then separated using Sephadex columns (G-50 and G-100); the phenol-sulfuric acid method was used to detect polysaccharides. According to the analysis results of HPGPC, five fractions (Fr. 2-2-1, Fr. 2-2-3, Fr. 2-2-4, Fr.2-2-5, Fr. 2-2-6) with different molecular weights were obtained. The five fractions were then further purified by DEAE-52 column and eluted using pure water and NaCl solution with increasing concentrations (0.05 M, 0.1 M, 0.2 M, 0.3 M, 0.5 M). Using the phenol-sulfuric acid method, the eluates with various solvents were collected. The water-eluted fractions of Fr. 2-2-3, Fr. 2-2-4, Fr. 2-2-5, and Fr. 2-2-6 were collected respectively and lyophilized to obtain MCPa (98 mg), MCPb (142 mg), MCPc (56 mg) and MCPd (44 mg).

### Determination of total sugar content

The content of total sugar in MCPs was determined by the phenol-sulfuric acid method [21]. A standard curve was generated using a group of accurate Glc reference solutions of varying concentrations (0, 1, 10, 50, 100, and 150 *µ*g/mL). In test tubes, 1 mL of each standard solution was added, followed by 1 mL of a 5% phenol solution and 5 mL of concentrated sulfuric acid. The absorbance of the mixtures was measured at 490 nm. To determine sample polysaccharides content, 80 *µ*g/mL solution of each sample was prepared. Each test tube contained exactly 1 mL of each sample solution, which was treated in the same manner as the standard products. We substituted the absorbance of samples in the standard curve to calculate the content of total sugar.

### Determination of uronic acid content

Uronic acid content of MCPs was determined by the carbazole-sulfuric method [22]. GalA was used as a standard. A group of standard solutions with various concentrations (0, 1, 10, 30, 80, 100 *µ*g/mL) was accurately prepared. In test tubes, 1 mL of each standard solution was measured, 5 mL of concentrated sulfuric acid was added, and the mixture was heated at 85 ℃ for 20 min. After cooling to room temperature, 200 *µ*L of carbazole ethanol solution was added to each test tube and heated at 100 ℃ for 15 min. Then we measured the absorbance at 520 nm and made a standard curve. The samples were accurately weighed and respectively prepared into a solution of 200 *µ*g/mL, and the absorbance was then determined by the same method as above. The absorbance of samples was substituted in the standard curve to calculate the content of uronic acid.

### Determination of protein content

The content of protein in MCPs was determined by the Bradford method [23]. We dissolved 0.1 g of Coomassie brilliant G-250 in 50 mL of 95% ethanol, added 100 mL of 85% phosphoric acid, and then pure water was added to 1000 mL to obtain Coomassie brilliant G-250 staining solution. Using BSA as a standard, a series of accurate reference solutions of different concentrations (0, 1, 10, 30, 80, 100 *µ*g/mL) were prepared. We accurately measured 1 mL of each standard solution in test tubes, added 5 mL of Coomassie brilliant G-250 staining solution, shook the mixture well, and allowed it to stand for 2 min. The absorbance was measured at 595 nm. To determine sample protein content, each sample was prepared into a solution of 200 *µ*g/mL. The test tubes contained exactly 1 mL of sample solution and were treated in the same manner as the standard products. The absorbance of samples was substituted in the standard curve to determine the protein content.

### Determination of molecular weight

The molecular weights of MCPs were determined by HPGPC [35]. A series of dextran standards with known molecular weights of 2700, 5250, 9750, 13,050, 36,800, 646,500, 135,350 and 300,600 Da was diluted with pure water respectively to prepare standard solutions of 1 mg/mL. The standard solutions were determined by HPGPC to draw a retention time-molecular weight standard curve. The samples were dissolved in pure water to 1 mg/mL and analyzed by HPGPC in the same way as standards. The retention time was substituted in the standard curve to calculate the molecular weights of samples.

### UV spectroscopy

The samples were dissolved in pure water to 1 mg/mL, and were detected by a UV–visible spectrophotometer (UV-2401PC, Shimadzu, Japan) in the wavelength range of 190–800 nm.

### Determination of the monosaccharide composition

The monosaccharide composition of MCPs was determined using HPLC analyses of the PMP derivatives obtained from the samples [36]. In order to prepare a mixed standard solution with a concentration of 1 mg/mL, we precisely weighed 1.5 mg of each monosaccharide standard including Man, Rha, Ara, Glc, Gal, GlcA, GalA, GlcNAc and Fuc, then mixed the standards and dissolved them in 1.5 mL of pure water. We measured 1 mL of the mixed standard solution in a hydrolysis tube, added 2 mL of 4 M TFA, sealed, and heated it at 110 ℃ for 2 h. The solution was then dried in a test tube by a rotary evaporator. About 2 mL of methanol was added thrice to remove the residual TFA. The dried hydrolysis product was then dissolved in 200 *µ*L of pure water, added with 200 *µ*L of 0.6 M NaOH and 400 *µ*L of 0.5 M PMP-methanol solution, and heated at 70 ℃ for 1 h. After cooling to room temperature, the product was analyzed by HPLC. We used an HPLC apparatus (LC 20-AT, Shimadzu, Japan) equipped with a UV-Vis detector (SPD-20 A, Shimadzu, Japan) and a tandem C18 reversed-phase column (Hadesil C18-Bio, Alcoen, United Kingdom). The column temperature was 25 ℃, the flow rate was 1 mL/min, the analysis time was 60 min, and the detection wavelength was 250 nm. The mobile phase A was 0.1 M ammonium acetate solution and the mobile phase B was acetonitrile. The concentration of the mobile phase B gradient changed from 17% (0–30 min) to 20% (33–60 min). The samples were respectively measured to prepare solutions of 1 mg/mL, hydrolyzed by TFA, derivated by PMP and analyzed by HPLC in the same way as mixed standards. The monosaccharide composition of MCPs was determined according by comparing the HPLC spectrograms of mixed monosaccharide standards and the samples.

### IR spectroscopy

About 1 mg of each sample was weighed, mixed with KBr and compressed into a tablet. The IR spectrum was recorded by an IR spectrometer (Tensor-27, Bruker, Germany) and the scanning wavelength range was 450–4000 cm^− 1^.

### Methylation analyses

The methylation–acetylation derivatives of MCPs were analyzed by HPLC to detect the linkage types contained. The reactions were carried out according to previous studies with some modifications [37]. In a test tube, 5 mg of each sample was dissolved in 1 mL of anhydrous DMSO. Then 20 mg of NaOH and 500 *µ*L of iodomethane were added, and reacted ultrasonically under the dark for 1 h and continued with stirring reactions for 7 h. We repeated the methylation reaction three times and terminated it by adding 2 mL of pure water. Next, the products were extracted thrice by CHCl_3_, and the organic layer was collected and dried by a rotary evaporator. After that, the residue was dissolved in pure water, dialyzed with a molecular weight cutoff of 3 kDa and lyophilized. The obtained methylation products were then hydrolyzed by 1.5 mL of 2 M TFA at 110 ℃ for 2 h, and dried by a rotary evaporator. About 2 mL of methanol was added thrice to remove the residual TFA. After acid hydrolysis, 25 mg of NaBD_4_ and 4 mL of NaOH solution (pH = 10) were added and stirring reacted at 50 ℃ for 2 h. The reduction reaction was terminated with 125 *µ*L of glacial acetic acid and dried by a rotary evaporator. The dried products were then acetylated with 500 *µ*L of pyridine and 1 mL of acetic anhydride at 100 ℃ for 1 h and the reaction was terminated by the addition of 1 mL of water. After cooling to room temperature, the methylation-acetylation products were extracted by CH_2_Cl_2_ and the organic layer was collected and dried by a rotary evaporator to about 200 *µ*L. After that, the products were analyzed by GC-MS using a GC system (7890 A, Agilent, USA) with a paired inert mass selective detector (5957 C, Agilent, USA) and a tandem column (DB-5MS, Agilent, USA). The carrier gas was helium and the flow rate was 1.5 mL/min. The split flow was 5:1. The column temperature started from 100 ℃ for 5 min, increased to 250 ℃ at 5 ℃/min and held. The ionization source was an electron bombardment source and the scan mode were full scan. The analysis time was 40 min.

### NMR spectroscopy

About 25 mg of each sample was measured and dissolved in 500 *µ*L of D_2_O. The spectra of 1D NMR (^1^H NMR, ^13^C NMR) and 2D NMR (COSY, TOCSY, ROESY, HSQC, HMBC) were recorded by an NMR spectrometer (Ascend 800 MHz, Bruker, Germany) and 3-trimethylsilyl-(2,2,3,3-^2^H4)-propionic acid sodium (TSP) was used as an internal.

### Inhibitory activity on *α-*glucosidase

The *α-*glucosidase inhibitory activity test was carried out according to a previous study of our research team [38].

## Supplementary Information

**Additional file 1.** Supplementary tables and figures.

## Data Availability

The data that support the findings of this study were available on request from the corresponding author, upon reasonable request.
