# Supplementary material for: Structural characterizations and α-glucosidase inhibitory activities of four Lepidium meyenii polysaccharides with different molecular weights
Source: Nat Prod Bioprospect. 2023 Jun 6;13(1):18. doi: 10.1007/s13659-023-00384-1 (PMC10244309; doi:10.1007/s13659-023-00384-1)
Supplement: Supplementary file 1 — Additional file 1. Supplementary tables and figures. [file 13659_2023_384_MOESM1_ESM.docx]

**Additional file 1**

**Structural characterizations and *α*-glucosidase inhibitory activities of four *Lepidium meyenii* polysaccharides with different molecular weights**

Luan Wen^1,2,†^, Zhou-Wei Wu^1,2,†^, Li-Wu Lin^1,2^, Abdulbaset Al-Romaima^1,2^, Xing-Rong Peng^1,2^, and Ming-Hua Qiu^1,2,*^

^1^ State Key Laboratory of Phytochemistry and Plant Resources in West China, and Yunnan Key Laboratory of Natural Medicinal Chemistry, Kunming Institute of Botany, Chinese Academy of Sciences, Kunming 650201, Yunnan, People’s Republic of China

^2^ University of Chinese Academy of Sciences, Beijing 100049, People’s Republic of China

^†^Luan Wen and Zhou-Wei Wu contributed equally to this work

^*^Corresponding author

Tel: +86-0871-65223327

Fax: +86-0871-65223325

E-mail: mhchiu@mail.kib.ac.cn

**Contents of Additional file 1**

[**Table S1** Concentration-absorbance standard curves of content determination of total sugar, uronic acid and protein 1](#_Toc136335304)

[**Fig. S1** HPGPC analysis of dextran standards STD1-8 1](#_Toc136335305)

[**Fig. S2** Retention time-molecular weight standard curve in HPGPC analysis 2](#_Toc136335306)

[**Fig. S3** HPGPC analyses of MCPs 2](#_Toc136335307)

[**Fig. S4** Molecular weight distributions of MCPa (**A**); MCPb (**B**); MCPc (**C**); MCPd (**D**) 3](#_Toc136335308)

[**Fig. S5** UV spectrums of MCPs 3](#_Toc136335309)

[**Fig. S6** Monosaccharide composition analyses of MCPs. Man, mannose; Rha, rhamnose; GlcNAc, N-acetyl-glucosamine; GlcA, glucuronic acid; GalA, galacturonic acid; Glc, glucose; Gal, galactose; Ara, arabinose; Fuc, fucose 4](#_Toc136335310)

[**Fig. S7** IR spectra of MCPs 4](#_Toc136335311)

[**Fig. S8** GC chromatograms of methylation-acetylation products from MCPs 5](#_Toc136335312)

[**Fig. S9** Mass spectrums of 1,5-di-*O*-acetyl-1-deuterio-2,3,4,6-tetra-*O*-methyl-D-glucitol (**A**), 1,4,5-tri-*O*-acetyl-1-deuterio-2,3,6-tri-*O*-methyl-D-glucitol (**B**), 1,3,4,5-tetra-*O*-acetyl-1-deuterio-2,6-di-*O*-methyl-D-glucitol (**C**) and 1,4,5,6-tetra-*O*-acetyl-1-deuterio-2,3-di-*O*-methyl-D-glucitol (**D**) from MCPc 6](#_Toc136335313)

[**Fig. S10** ^1^H NMR spectrums of MCPs in D_2_O (800 MHz) 7](#_Toc136335314)

[**Fig. S11** ^13^C NMR spectrums of MCPs in D_2_O (200 MHz) 8](#_Toc136335315)

[**Fig. S12** ^1^H NMR spectrum of MCPc in D_2_O (800 MHz) 9](#_Toc136335316)

[**Fig. S13** ^13^C NMR spectrum of MCPc in D_2_O (200 MHz) 9](#_Toc136335317)

[**Fig. S14** COSY spectrum of MCPc in D_2_O 10](#_Toc136335318)

[**Fig. S15** TOCSY spectrum of MCPc in D_2_O 10](#_Toc136335319)

[**Fig. S16** ROESY spectrum of MCPc in D_2_O 11](#_Toc136335320)

[**Fig. S17** HSQC spectrum of MCPc in D_2_O 12](#_Toc136335321)

[**Fig. S18** HMBC spectrum of MCPc in D_2_O 12](#_Toc136335322)

**Table S1** Concentration-absorbance standard curves of content determination of total sugar, uronic acid and protein

| **Project** | **Standard** | **Standard curve** | **R^2^** |
| --- | --- | --- | --- |
| Total sugar content | Glc | Y = 0.0093X + 0.0308 | 0.9994 |
| Uronic acid content | GalA | Y = 0.0152X + 0.0369 | 0.9998 |
| Protein content | BSA | Y = 0.0054X + 0.0184 | 0.9935 |


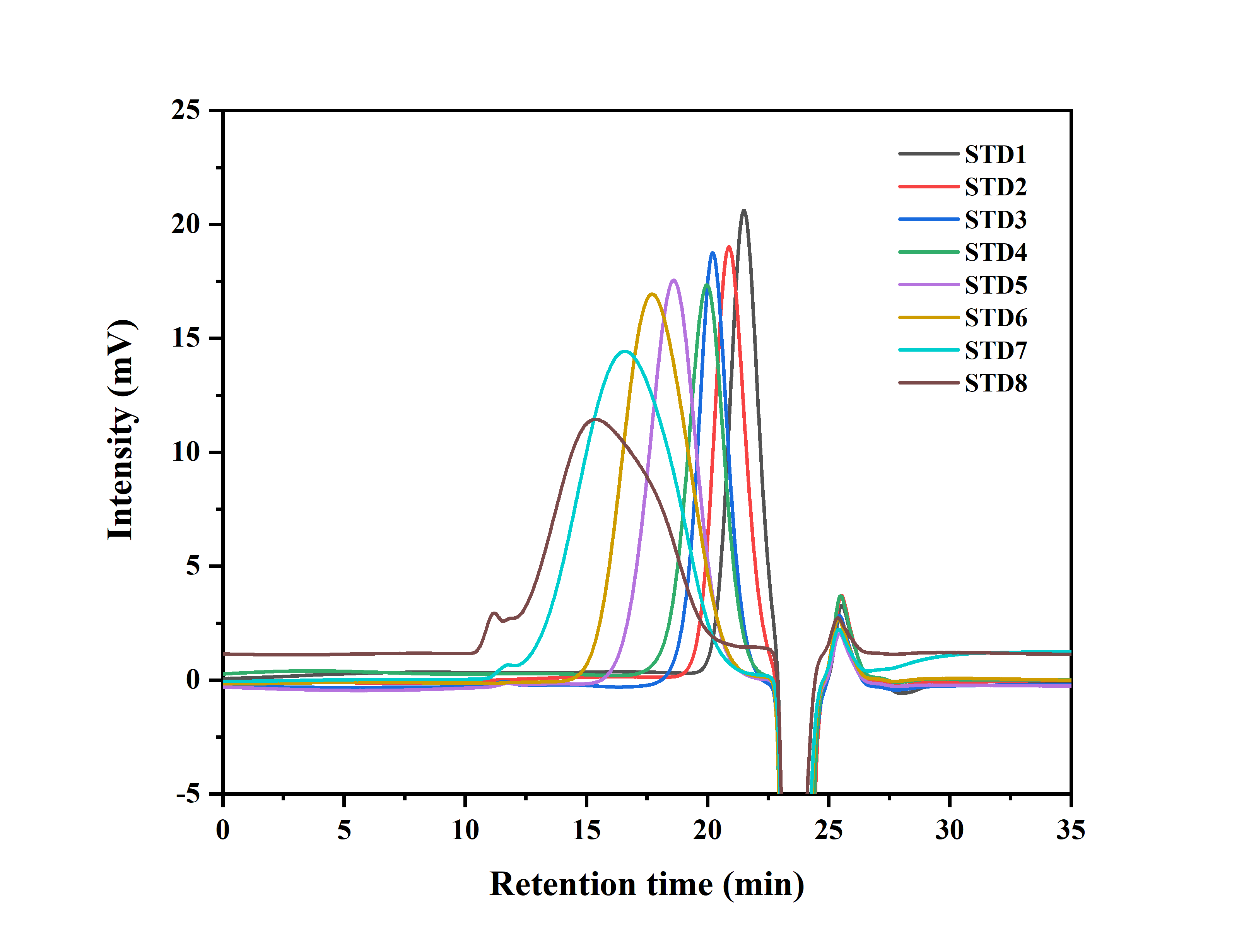


**Fig. S1** HPGPC analysis of dextran standards STD1-8


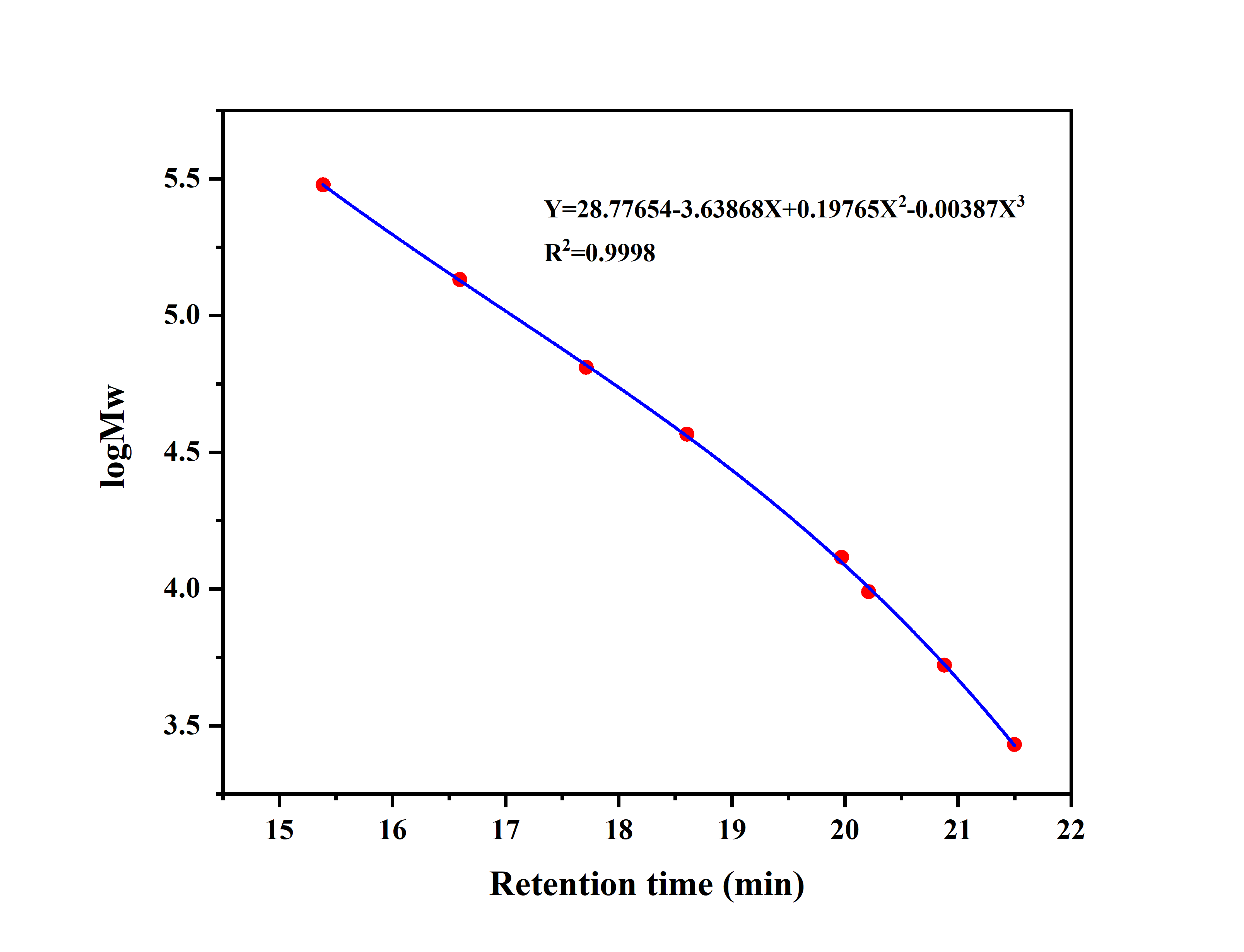


**Fig. S2** Retention time-molecular weight standard curve in HPGPC analysis


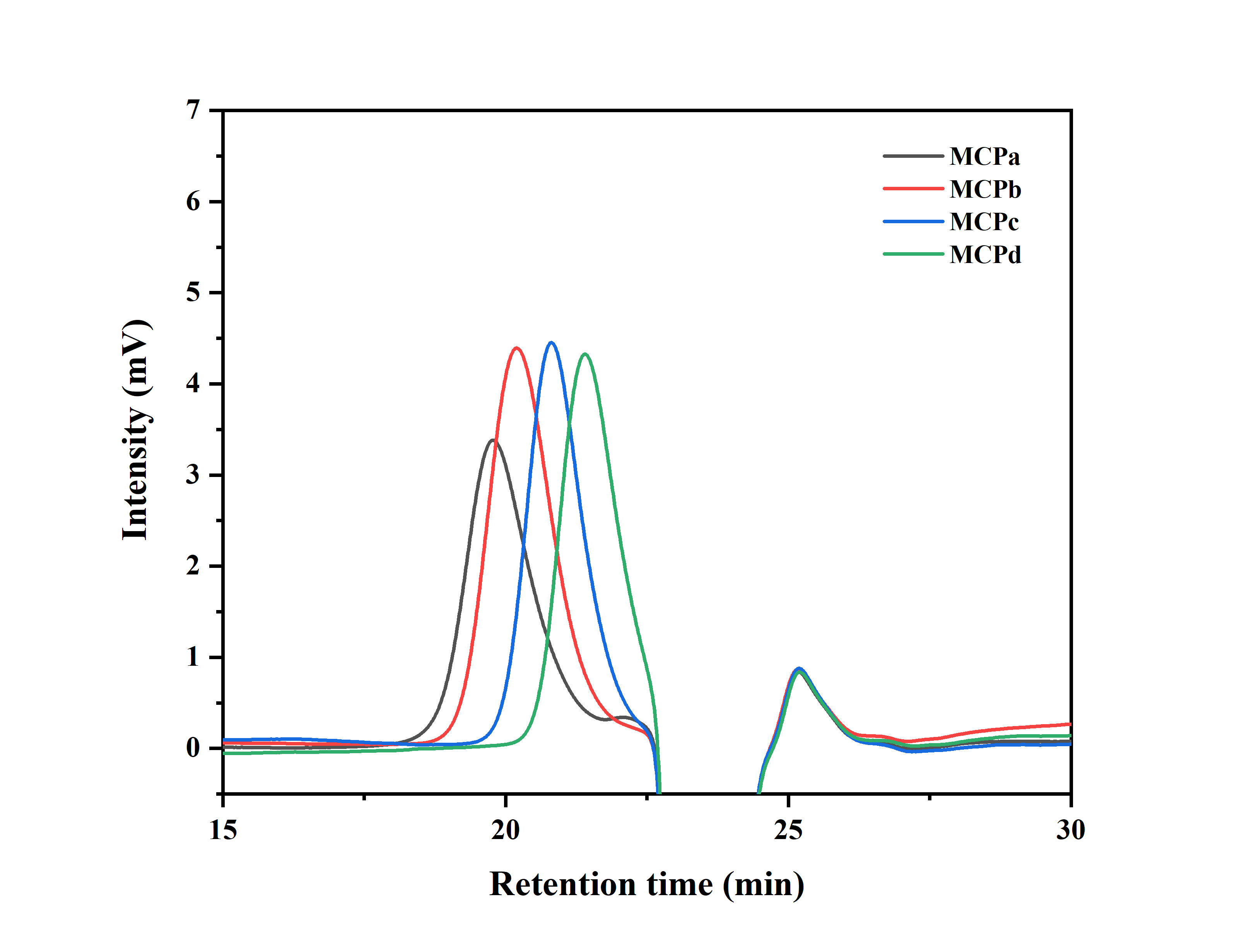


**Fig. S3** HPGPC analyses of MCPs


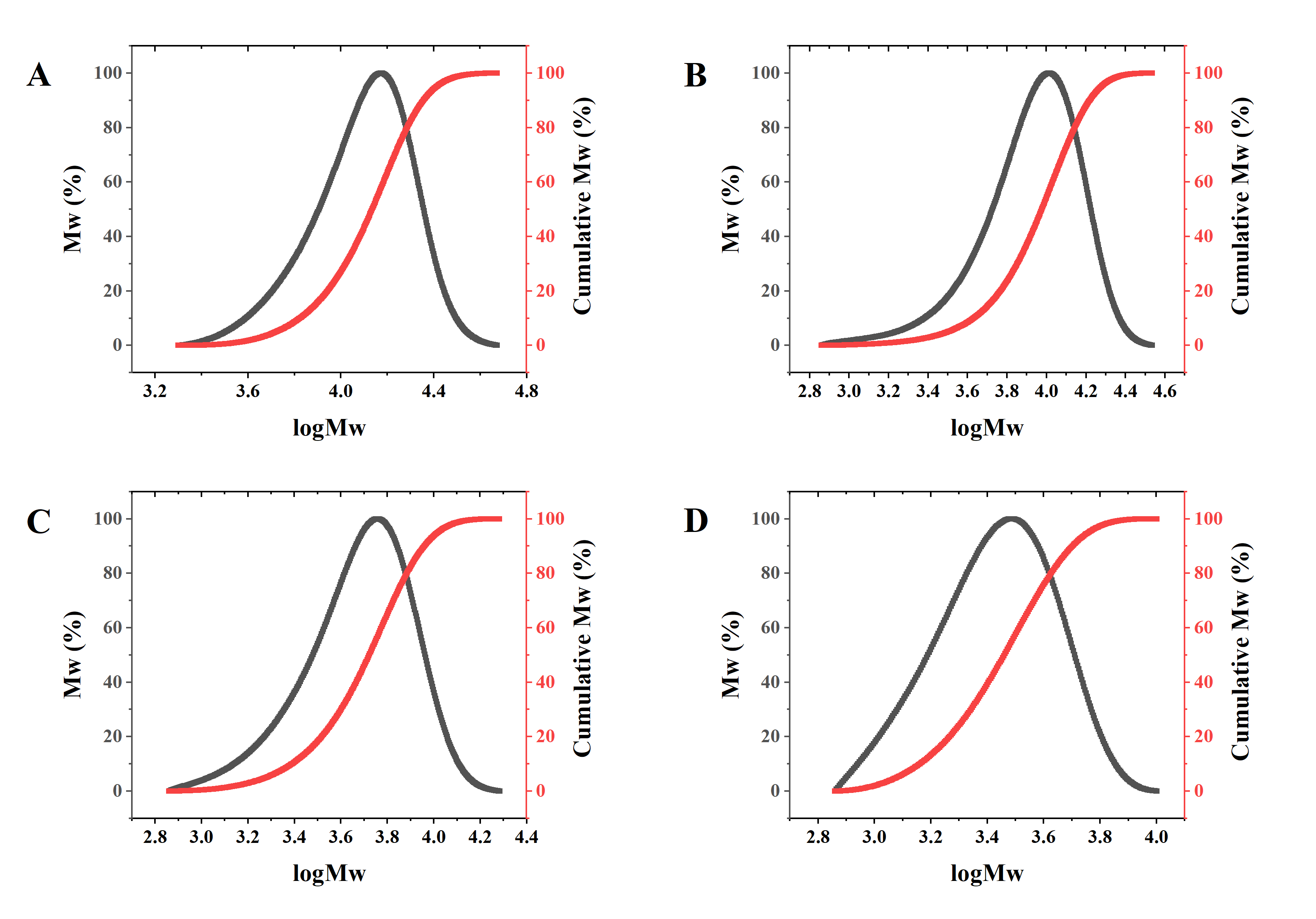


**Fig. S4** Molecular weight distributions of MCPa (**A**); MCPb (**B**); MCPc (**C**); MCPd (**D**)


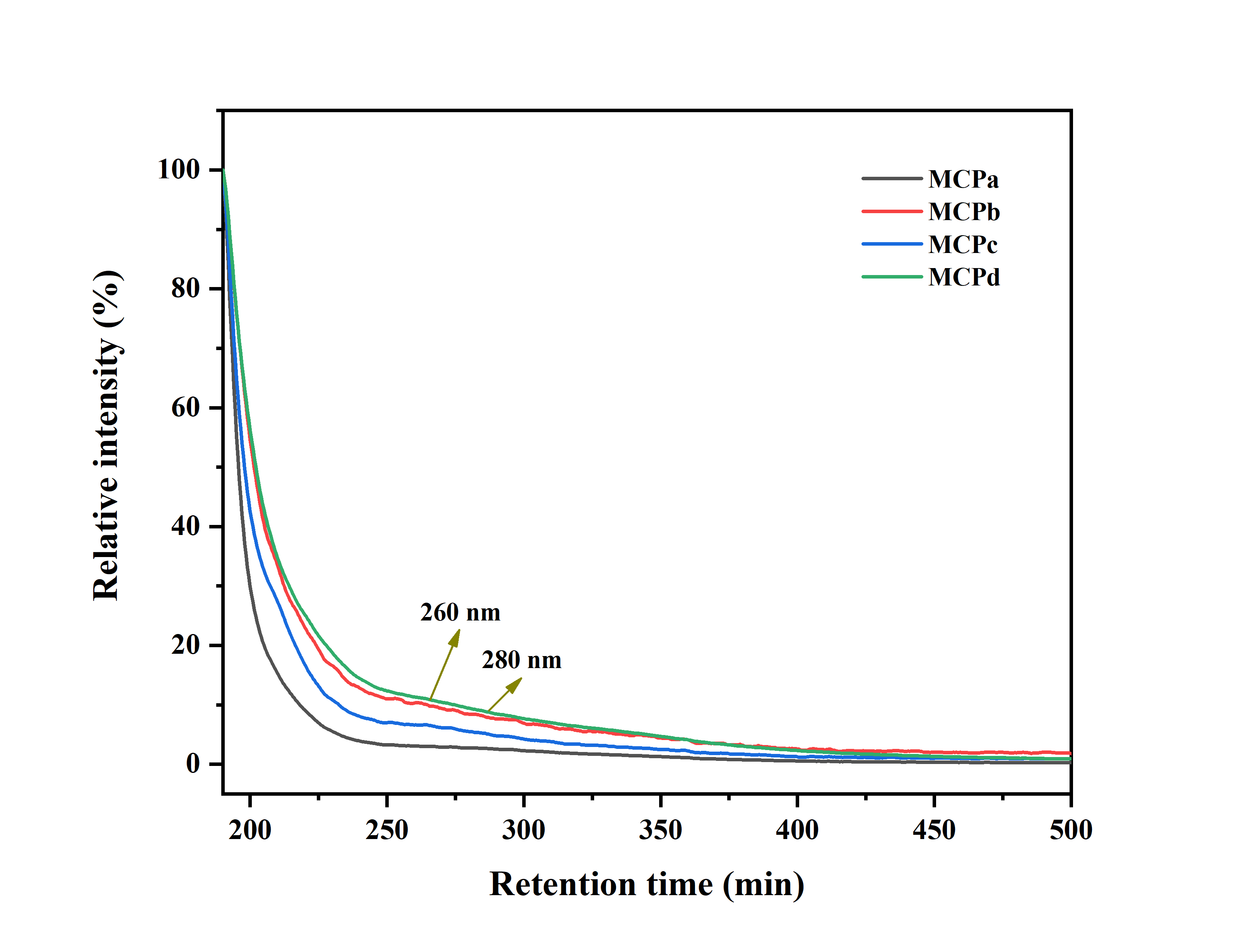


**Fig. S5** UV spectrums of MCPs


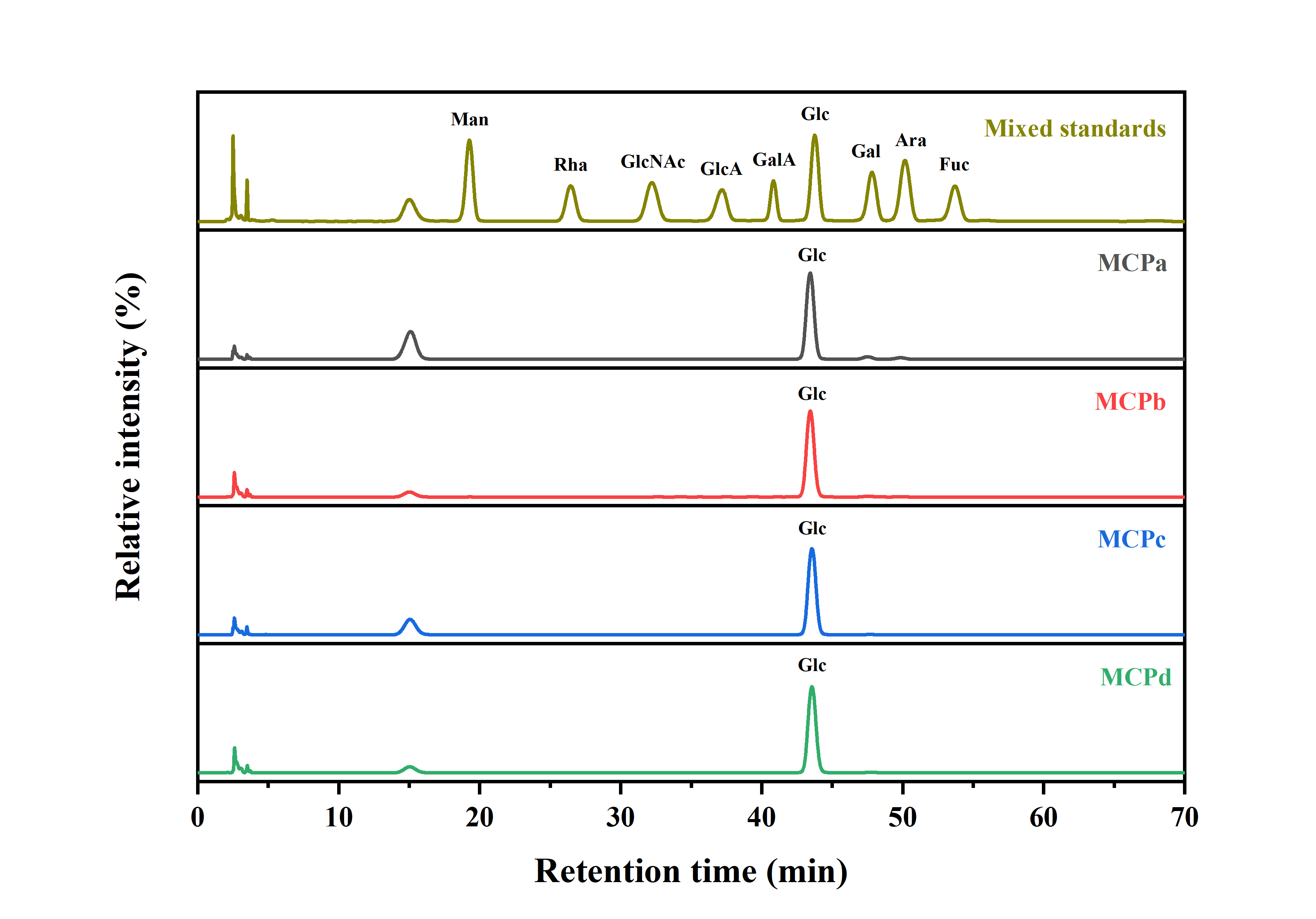


**Fig. S6** Monosaccharide composition analyses of MCPs. Man, mannose; Rha, rhamnose; GlcNAc, N-acetyl-glucosamine; GlcA, glucuronic acid; GalA, galacturonic acid; Glc, glucose; Gal, galactose; Ara, arabinose; Fuc, fucose


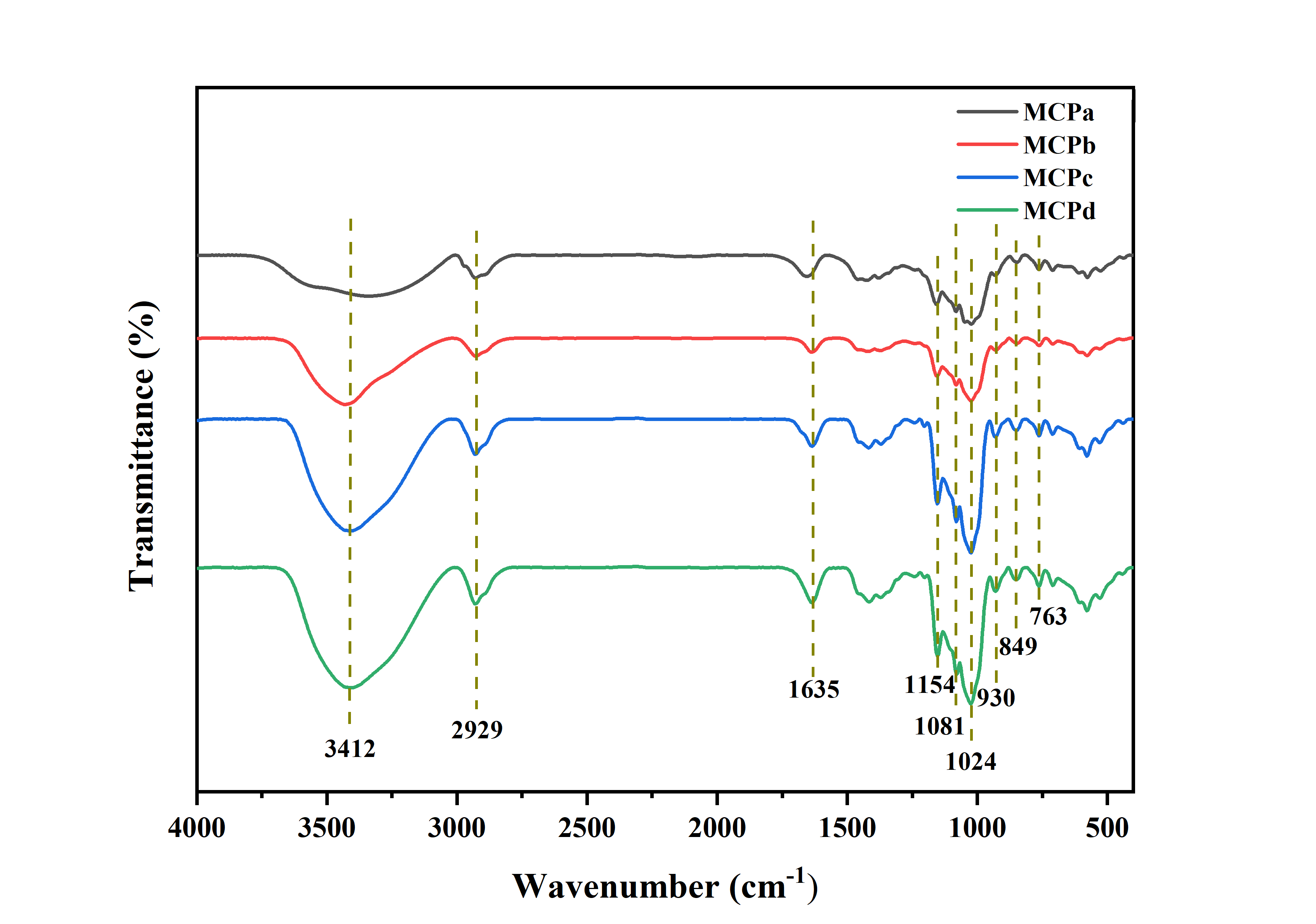


**Fig. S7** IR spectra of MCPs


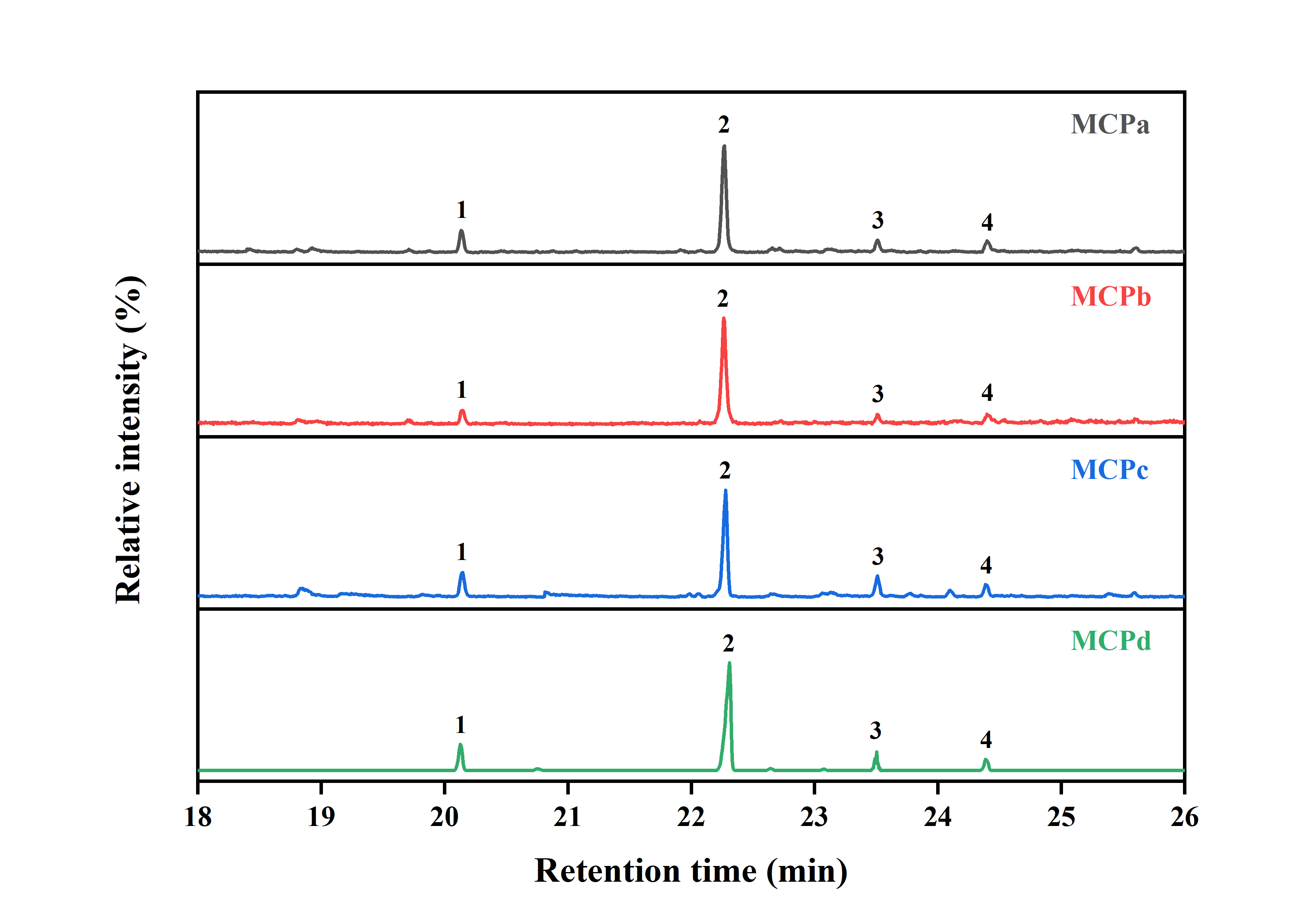


**Fig. S8** GC chromatograms of methylation-acetylation products from MCPs


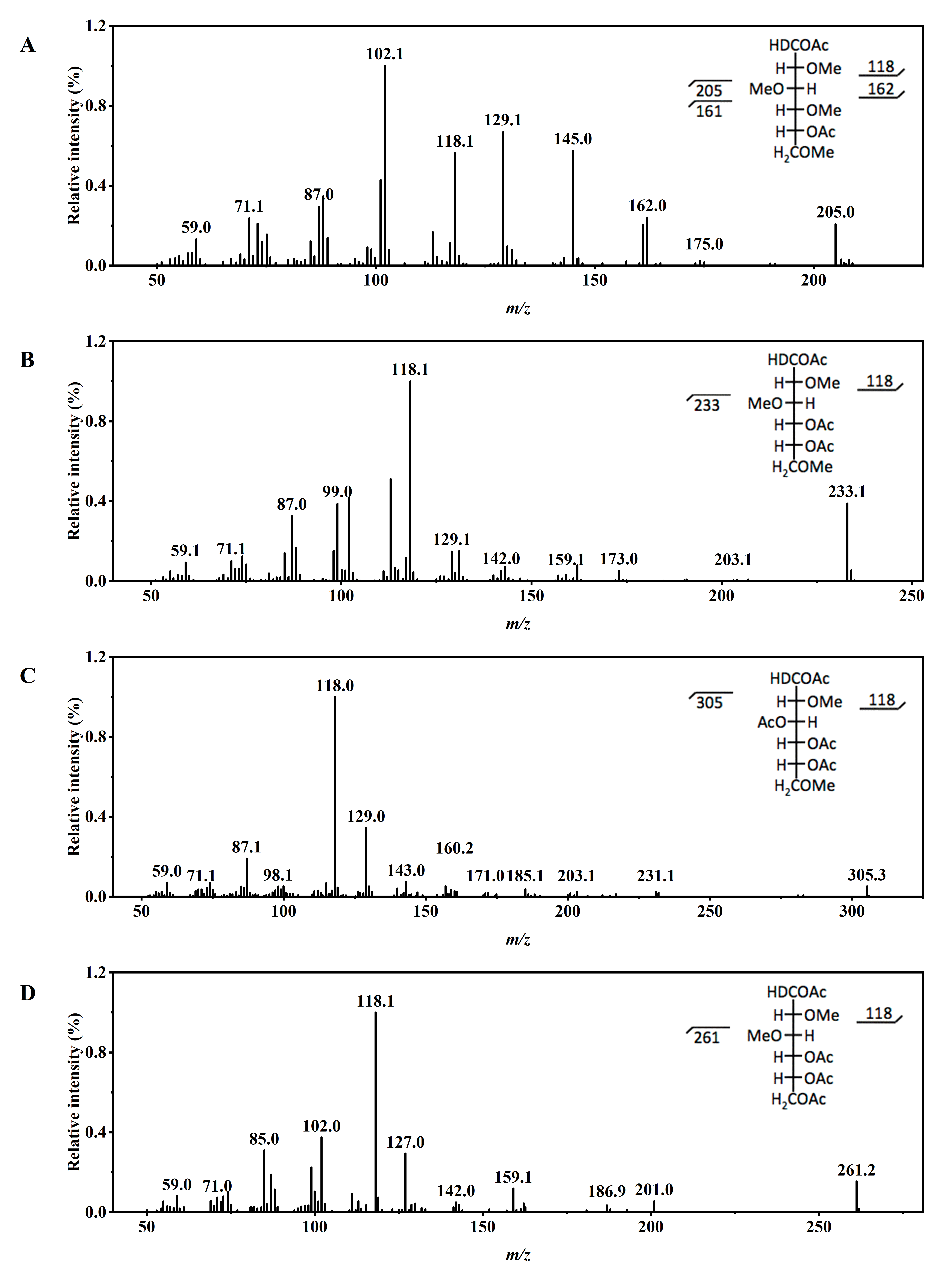


**Fig. S9** Mass spectrums of 1,5-di-*O*-acetyl-1-deuterio-2,3,4,6-tetra-*O*-methyl-D-glucitol (**A**), 1,4,5-tri-*O*-acetyl-1-deuterio-2,3,6-tri-*O*-methyl-D-glucitol (**B**), 1,3,4,5-tetra-*O*-acetyl-1-deuterio-2,6-di-*O*-methyl-D-glucitol (**C**) and 1,4,5,6-tetra-*O*-acetyl-1-deuterio-2,3-di-*O*-methyl-D-glucitol (**D**) from MCPc

**MCPa**

**MCPb**

**MCPc**

**MCPd**

**Fig. S10** ^1^H NMR spectrums of MCPs in D_2_O (800 MHz)

**MCPa**

**MCPb**

**MCPc**

**MCPd**

**Fig. S11** ^13^C NMR spectrums of MCPs in D_2_O (200 MHz)

**A/C3**

**B3/C6**

**A/B/D6**

**D4**

**D2**

**D3**

**A/B/C2**

**A/B/C4**

**A/B/C5**

**D5**

**D1**

**B/C1**

**A1**

**Fig. S12** ^1^H NMR spectrum of MCPc in D_2_O (800 MHz)

**A/B/D6**

**D4**

**C6**

**D5**

**D2**

**D3**

**A/B/C2**

**A/B/C5**

**A/C3**

**A/B/C4**

**B3**

**D1**

**A1**

**B/C1**

**Fig. S13** ^13^C NMR spectrum of MCPc in D_2_O (200 MHz)

**A/B/C (1, 2)**

**D (3, 4)**

**B (2, 3)**

**A/C (2, 3)**

**D (1, 2)**

**Fig. S14** COSY spectrum of MCPc in D_2_O

**D (6, 4)**

**D (3, 4)**

**D (2, 4)**

**A/C (3, 4)**

**A/C (3, 5)**

**A/B/C (5, 4)**

**D (1, 5)**

**A/B/C (1, 4)**

**A/B/C (1, 2)**

**D (1, 2)**

**A/B/C (1, 3)**

**Fig. S15** TOCSY spectrum of MCPc in D_2_O

**A**

**(A1, B/C4)**

**A (1, 6)**

**(B/C1, A4)**

**D (2, 4)**

**D (3, 4)**

**A/C (3, 5)**

**A/C (3, 2)**

**A/B/C (4, 5)**

**A/C (6, 4)**

**B/C (1, 2)**

**A (1, 3)**

**A (1, 2)**

**B**

**(D1, B3/C6)**

**D (1, 4)**

**Fig. S16** ROESY spectrum of MCPc in D_2_O

**A/C3**

**D2**

**A/B/C2**

**D1**

**A1**

**B/C1**

**A/B/C4**

**B3**

**D3**

**D4**

**A/B/C5**

**D5**

**C6**

**A/B/D6**

**Fig. S17** HSQC spectrum of MCPc in D_2_O

**D (6, 4)**

**D4**

**A/B/D6**

174 Hz

174 Hz

172 Hz

**A1**

**B/C1**

**(A/B/C4, A/B/C1)**

**(B3/C6, D1)**

**D1**

**D (4, 6)**

**D (3, 4)**

**D (4, 3)**

**A/B/C4**

**A/C (6, 4)**

**A/C (3, 4)**

**A/C3**

**A/C (3, 2)**

**D (1, 3)**

**D (1, 2)**

**B/C (1, 2)**

**C(1, 3)**

**B(1, 3)**

**(A1, A/B4)**

**A (1, 3)**

**A (1, 2)**

**Fig. S18** HMBC spectrum of MCPc in D_2_O
